# Blood-Based Detection of BRAF V600E in Gliomas and Brain Tumor Metastasis

**DOI:** 10.3390/cancers13061227

**Published:** 2021-03-11

**Authors:** Keiko M. Kang, Koushik Muralidharan, Anudeep Yekula, Julia L. Small, Zachary S. Rosh, Pamela S. Jones, Bob S. Carter, Leonora Balaj

**Affiliations:** 1 Department of Neurosurgery, Massachusetts General Hospital and Harvard Medical School, Boston, MA 02115, USA; keikok11@gmail.com (K.M.K.); kmuralidharan@mgh.harvard.edu (K.M.); AYEKULA@mgh.harvard.edu (A.Y.); julia.small12@gmail.com (J.L.S.); Zrosh@mgh.harvard.edu (Z.S.R.); psjones@partners.org (P.S.J.); 2 School of Medicine, University of California San Diego, San Diego, CA 92092, USA

**Keywords:** brain tumor, glioma, brain tumor metastasis, melanoma, liquid biopsy, cfDNA (cell free DNA), extracellular vesicles

## Abstract

**Simple Summary:**

The BRAF V600E mutation has been identified as a key driver in brain tumors and brain tumor metastasis. The ability to detect this mutation in a minimally invasive plasma assay offers advantages over traditional tissue-based biopsy for the disease diagnosis and monitoring. The aim of this study was to develop an assay for the detection of BRAF V600E in the plasma of patients with brain tumors and brain tumor metastasis. We demonstrate BRAF V600E detection using a novel plasma-based ddPCR assay. We detect the mutation in circulating nucleic acids in 4/5 patients with mutant gliomas and metastatic melanoma. We also show correlation between plasma BRAF V600E and clinical status. This proof of principle study is important in the context of application of liquid biopsy in plasma to the neuro-oncologic field. The assay may be useful as a diagnostic adjunct, prognostication tool, and method for monitoring of disease and treatment response.

**Abstract:**

Liquid biopsy provides a minimally invasive platform for the detection of tumor-derived information, including hotspot mutations, such as BRAF V600E. In this study, we provide evidence of the technical development of a ddPCR assay for the detection of BRAF V600E mutations in the plasma of patients with glioma or brain metastasis. In a small patient cohort (*n* = 9, *n* = 5 BRAF V600E, *n* = 4 BRAF WT, *n* = 4 healthy control), we were able to detect the BRAF V600E mutation in the plasma of 4/5 patients with BRAF V600E-tissue confirmed mutant tumors, and none of the BRAF WT tumors. We also provide evidence in two metastatic patients with longitudinal monitoring, where the plasma-based BRAF V600E mutation correlated with clinical disease status. This proof of principle study demonstrates the potential of this assay to serve as an adjunctive tool for the detection, monitoring, and molecular characterization of BRAF mutant gliomas and brain metastasis.

## 1. Introduction

Brain tumors, most commonly metastasis and gliomas, are a highly heterogeneous disease group with poor prognosis despite invasive and intensive multimodal treatment [1,2,3]. Diagnosis often involves imaging and tissue-based biopsy, and longitudinal disease monitoring currently relies on serial magnetic resonance imaging to assess treatment response, progression, or recurrence. However, imaging is limited in its detection of molecular heterogeneity and tumor evolution and obtaining tissue for histologic and molecular analysis is not always feasible due to the highly invasive nature of the surgery [4]. Liquid biopsy provides a minimally invasive platform for the detection of tumor-derived genomic and proteomic material in biofluids. Liquid biopsy-based strategies provide the ability to diagnose intracranial disease based on specific biomarkers, monitor response to treatment and disease progression, and provide real-time information on the molecular characteristics of the tumor [5,6,7,8]. While the detection of central nervous system (CNS) tumor hotspot mutations and molecular markers within the cerebrospinal fluid (CSF) of patients has been accomplished with high sensitivity and specificity [9,10,11,12], the detection of these targets in plasma has been difficult due to lower levels of circulating tumor nucleic acids, including cell-free DNA (cfDNA) and tumor-associated EV RNA (exRNA) [13]. In patients with primary cancers outside the CNS, liquid biopsy assays have been developed for the diagnosis and monitoring of several cancers, including malignant melanoma, colorectal cancer and prostate cancer [8,14,15,16].

The BRAF V600E mutation is of particular interest in neoplasia, as BRAF mutations can result in constitutive activation of the MAPK signaling pathway and contribute to uncontrolled cell proliferation. This oncogenic pathway is pivotal to several cancers, including melanoma, colorectal, and papillary thyroid cancer [17,18,19]. In the realm of primary CNS tumors, the BRAF V600E mutation has been implicated as a key driver for papillary craniopharyngiomas [20] and certain gliomas, such as pilocytic astrocytomas, pleomorphic xanthoastrocytoma (PXA), ganglioma, and GBM, most commonly epithelioid GBM [21,22,23]. Furthermore, the BRAF V600E is an actionable mutation. The advent of BRAF inhibitor therapies has revolutionized the field of metastatic melanoma and papillary craniopharyngiomas [24,25,26,27]. Their benefit in gliomas is currently under investigation [23].

In this paper, we focus on the technical development of the BRAF V600E hotspot mutations in gliomas and CNS metastases. In our cohort of 13 patients (*n* = 5 BRAF V600E, *n* = 4 BRAF WT, *n* = 4 healthy controls) we characterize the mutant allele frequency in all tumor tissues as well as the plasma samples from patients with confirmed BRAFV600E gliomas. Further, in two patients with metastatic disease and available longitudinal samples, we determine the BRAF V600E plasma levels by the ddPCR assay and correlate the findings with clinical status.

## 2. Results

### 2.1. Optimization of BRAF V600E and BRAF WT ddPCR Assay for cfDNA and EV RNA/DNA

**Figure 1:**
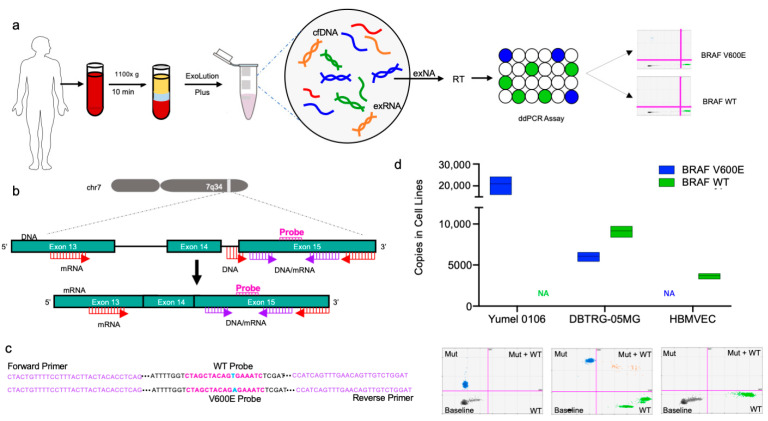
BRAF V600E and BRAF Wild Type ddPCR Assay overview and design. (**a**) Schematic depicting workflow of ddPCR Assay, from blood collection, extraction of exNA using ExoLution Plus Kit (Exosome Diagnostics), reverse transcription, and ddPCR to sample readout; (**b**) Diagram depicting characteristics of primer sets and overall assay design. BRAF DNA primers bind to exon 15 and the adjacent intron. BRAF mRNA primers lie within exon 13. The universal primer set, detects both DNA and mRNA and lies within exon 15. Primer binding sites for DNA and mRNA are indicated in red, while universal primer binding sites are indicated in purple; (**c**) Sequences for the BRAF V600E and BRAF wild type probes (pink), and universal primer sequence (purple) are also denoted. The BRAF V600E point mutation (1700 T>A) is highlighted in blue; (**d**) Absolute quantification of BRAF mutant and BRAF wild type copies (*y*-axis) of different cell lines (*x*-axis) are graphed. The three cell lines tested, ordered left to right on the graph, were Yumel 0106 (homozygous for BRAF V600E), DBTRG-05MG (heterozygous for BRAF V600E), and HBMVEC (BRAF wild type). ddPCR 2D amplitude plots are shown for each cell line.

In this study, we have developed a ddPCR assay to detect the BRAF V600E mutation in circulating plasma-derived cell free DNA and EV RNA. The overall assay workflow is shown in Figure 1a. BRAF p.V600E (c.1799 T>A) lies within exon 15 of chromosome 7 and can thus be detected at both the DNA and mRNA level. In order to enhance detection of the mutation, we designed and tested multiple primer sets to detect the mutation in DNA, mRNA, as well as both DNA and mRNA targets (Figure 1b). Exact sequences for the BRAF V600E and wild type probes as well as the universal primer set are shown in Figure 1c.

Next, we studied the specificity of the assay to the BRAF V600E and BRAF WT sequences in cell lines (Figure 1d). Three cell lines, Yumel 0106 (homozygous for BRAF V600E), DBTRG-05MG (heterozygous for BRAF V600E), and HBMVEC (homozygous for BRAF WT) were tested. We isolated RNA from each cell line and 10 ng of cDNA was used as template for the universal primer set in replicates of 3–4 for absolute quantification of BRAF V600E and BRAF WT copies. In Yumel 0106 cell line, an average of 20,900 copies of BRAF V600E were detected, and an average of one copy of BRAF WT was detected. In DBTRG-05MG, an average of 6047 BRAF V600E and 9160 BRAF WT copies were detected. In HBMVEC cell line, an average of 3700 BRAF WT and three BRAF V600E copies were detected. The corresponding ddPCR 2D amplitude plots demonstrating separation of BRAF V600E and BRAF WT populations for each cell line are shown (Figure 1d)

**Figure 2:**
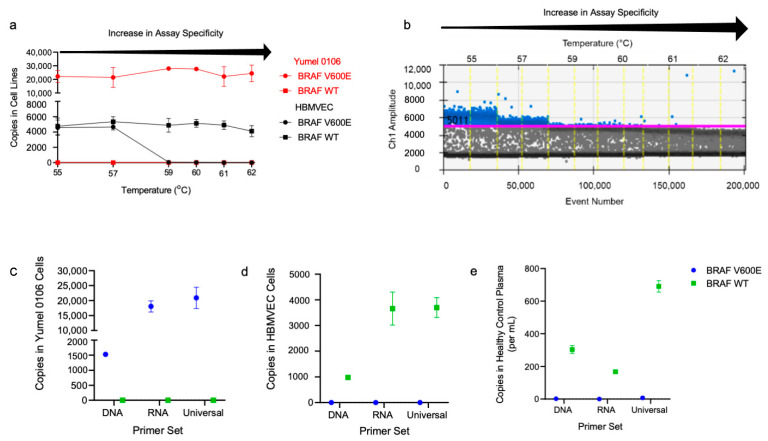
Optimization of the BRAF V600E and BRAF Wild Type ddPCR assay. (**a**) Absolute quantification of BRAF V600E (circle) and BRAF wild type (square) copies using the universal primer set in cDNA isolated from Yumel 0106 (red) and HBMVEC (black) cell lines are graphed with respect to temperature. Specificity of the assay increases with increasing annealing temperature, as shown by the arrow above the graph; (**b**) ddPCR 1D amplitude plot of HBMVEC cell line is shown. Mutant channel amplitude (*y*-axis) with respect to event number (lower *x*-axis) and temperature (upper *x*-axis) is shown. Gates (pink line) were set with respect to wild type background population. Positive events, shown as blue droplets above the gate, decrease as the annealing temperature increases. Absolute quantification of BRAF V600E and BRAF WT copies and copies per mL of plasma were assessed in (**c**) Yumel 0106, (**d**) HBMVEC, and (**e**) healthy control plasma using DNA, mRNA, and universal primer sets and plotted as mean +/− SD.

**Figure A1:**
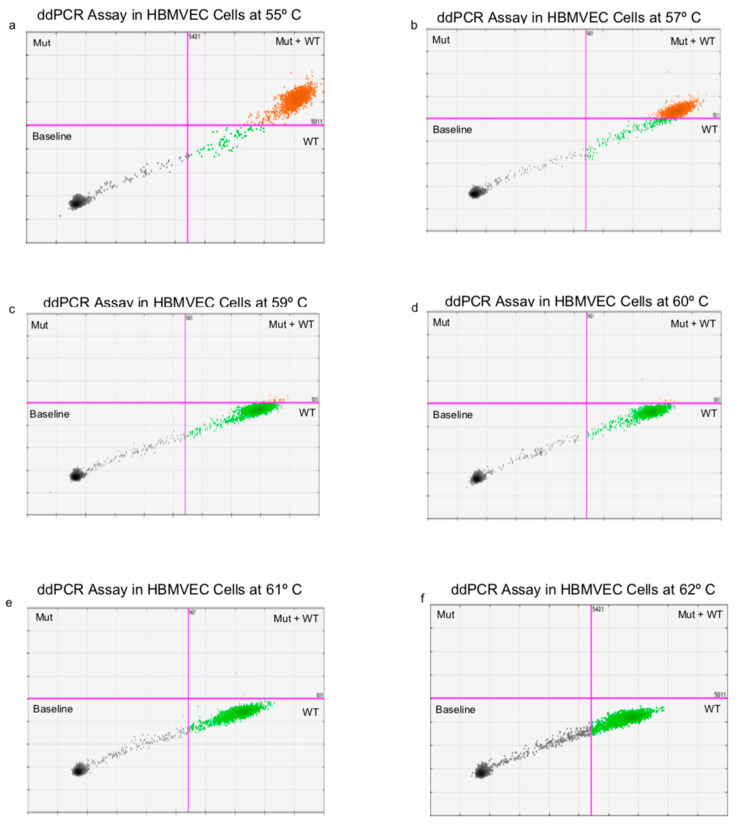
ddPCR 2D Plots of Temperature Gradient in HBMVEC Cell Line. Plots with wild type channel amplitude (*x*-axis) and mutant channel amplitude (*y*-axis) at 55 (**a**), 57 (**b**), 59 (**c**) 60 (**d**), 61 (**e**), and 62 °C (**f**) in HBMVEC cells with the universal primer are shown.

We then performed a temperature gradient to determine the optimal annealing temperature and reduce non-specific probe binding. Then, 10 ng of cDNA from HBMVEC and Yumel 0106 cell lines was used with the universal primer set and tested over a range of annealing temperatures from 55–62 °C. At lower annealing temperatures, we observed non-specific probe binding, resulting in false positive BRAF V600E detection in the wildtype HBMVEC cell line. At 55 °C, an average of 4567 false positive BRAF V600E copies were detected, which decreased to ~3 at an annealing temperature of 62 °C. The corresponding ddPCR 2D plots across these temperatures are shown in Figure A1. The expected BRAF V600E copies in the mutant Yumel 0106 cell line was, on average, 23,627 across all temperatures, and the expected BRAF WT copies in the wild type HBMVEC was also consistent, with an average of 4833 copies across all temperatures (Figure 2a). ddPCR 1D amplitude plot of the mutant channel in the HBMVEC cell line is shown, demonstrating a decrease in the false positive mutant droplets and merging of false positive and background populations below the gate (Figure 2b).

Each of the developed primer sets (DNA, mRNA, and universal) were then tested in Yumel 0106 (Figure 2c) and HBMVEC cell lines (Figure 2d), as well as in healthy control plasma (Figure 2e) in replicates of 2–4 to determine the assay that would allow for maximal detection of of BRAF copies. In the Yumel 0106 cell line, the universal primer set detected higher BRAF V600E copies (20,900) as compared to the DNA (1526, *p* = 0.002) or mRNA (18,040, *p* > 0.05) primer sets. In the HBMVEC cell line, the universal primer set detected higher BRAF WT copies (3700) as compared to the DNA (982, *p* = 0.007) or mRNA (3660, *p* > 0.05) primer sets. In healthy control plasma, the universal primer set detected significantly higher BRAF WT copies (690) than the DNA (303, *p* = 0.01) or mRNA (168, *p* = 0.03) primer sets. Highest detection of combined cfDNA and exRNA, here termed extracellular nucleic acid (exNA), was achieved using the universal primer set, which we used for all subsequent plasma sample analysis.

### 2.2. Determining Lower Limits of Blank and Lower Limits of Detection

**Figure 3:**
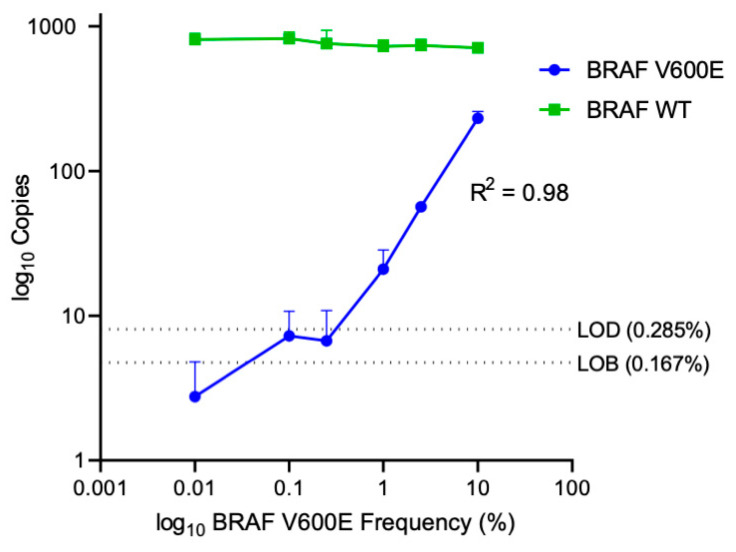
BRAF V600E serial dilutions in a constant background of BRAF WT. Log of absolute copy number of BRAF V600E (blue) and BRAF WT (green) are plotted against log mutant frequency. Data points are plotted as mean +/− SD. Serial dilutions of Yumel 0106 gDNA were prepared in a constant background of HBMVEC gDNA from 10% to 0% mutant allele frequency to determine the limit of detection and limit of blank of the ddPCR assay. The limit of blank (LOB), the highest apparent BRAF V600E concentration expected to be found in a sample without mutant input, and limit of detection (LOD), the concentration of BRAF V600E that can be reliably distinguished from background, are denoted by the dotted lines.

**Figure A2:**
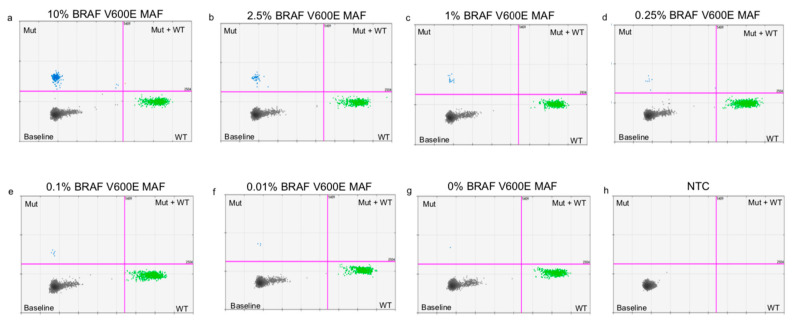
ddPCR 2D Plots of BRAF V600E serial dilutions in a constant background of BRAF WT. Plots of wild type channel amplitude (*x*-axis) and mutant channel amplitude (*y*-axis) of serial dilutions of BRAF V600E from Yumel 0106 cell lines in decreasing concentration from 10% MAF (**a**), 2.5% (**b**), 1% (**c**), 0.25% (**d**), 0.1% (**e**), 0.01% (**f**), to 0% MAF (**g**) into a background of BRAF WT from HBMVEC cell lines. The no template control (NTC) is also shown (**h**).

In order to determine the lower limit of blank (LOB) and detection (LOD) for the BRAF V600E and BRAF WT ddPCR assay with the universal primer set, we tested serial dilutions of mutant Yumel 0106 gDNA in a constant background of wild type HBMVEC gDNA. Assay input was held constant at 10 ng/µL of gDNA with a mutant allele frequency of 0.01–10%, in replicates of five. The limit of the blank, or the apparent frequency of BRAF V600E detected in a pure wild type sample, was found to be 4.73 copies/20 µL well. The limit of detection, or the lowest frequency of BRAF V600E that can be reliably distinguished from a pure wild type sample was found to be 8.08 copies/20 µL well [28]. This correlated to a lower limit of detection of 0.285% mutant allele frequency (Figure 3). In this paper, we define the limit of quantitation (LOQ) for the BRAFV600E assay as 0.285% mutant allele frequency (MAF), equivalent to the lower limit of detection. The corresponding ddPCR 2D amplitude plots for each serial dilution are shown in Figure A2.

### 2.3. Detection of BRAF V600E in Patient Tumor Tissue and Patient Plasma

**Figure 4:**
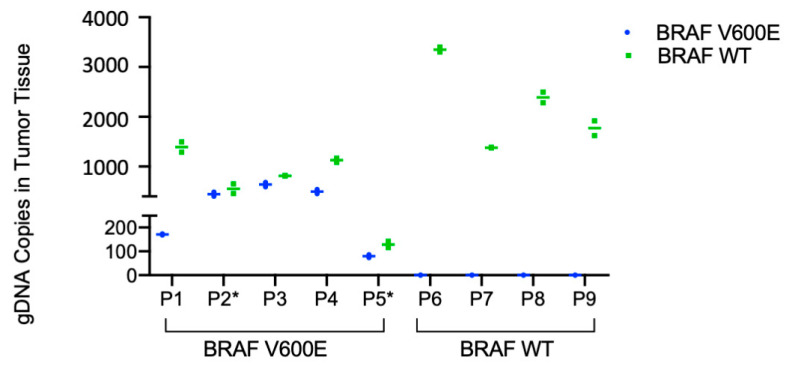
BRAF V600E and BRAF Wild Type ddPCR assay in patient tumor tissue. Absolute quantification of the BRAF V600E and wild type copy number in tumor tissue is plotted as mean +/− SD against patient study ID, grouped as known BRAF mutant and BRAF wild type as determined by either SNapShot assay or IHC. gDNA was isolated from fresh frozen tumor tissue or FFPE slides (*), and 10 ng was used as input for the ddPCR assay using the DNA primer set (ddPCR Tumor Assay).

**Figure A3:**
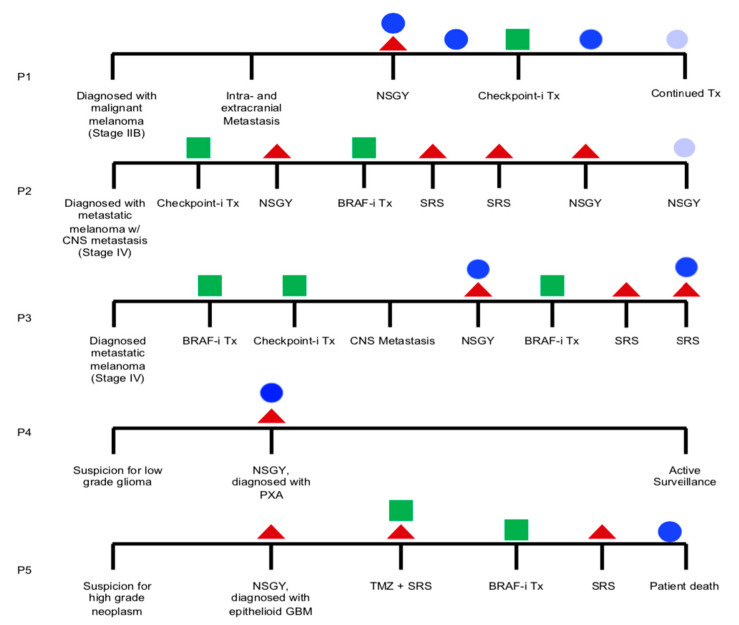
Clinical course of patients with BRAF V600E mutant tumors. For each patient, P1–P5, with a BRAF V600E mutant tumor, the clinical course is shown. Interventions including neurosurgery and stereotactic radiosurgery are denoted by a red triangle. Initiation of chemotherapy is denoted by a green square. Assay timepoints are denoted by circles. Plasma positive for BRAF V600E are denoted by dark blue circles while plasma negative for BRAF V600E are denoted by light blue circles.

Next, the BRAF V600E and BRAF WT assay was tested in patient tumor and plasma samples. Baseline patient characteristics are shown in Table 1 and the general clinical course of each patient is shown in Figure A3. Given the abundance of DNA in tumor tissue, the BRAF V600E and BRAF WT ddPCR assay with the DNA primer set (i.e., ddPCR Tumor Assay) was used to test patient tumor samples. Fresh frozen tumor tissue or FFPE slides were available for testing for all patients (*n* = 5 BRAF V600E, *n* = 3 fresh frozen tissue, *n* = 2 FFPE slides; *n* = 4 BRAF WT fresh frozen tissue). Then, 10 ng of gDNA was used as template and samples were run in duplicates. Among patients with known BRAF V600E mutant tumors, absolute BRAF V600E copy number in tumor tissue ranged from 80–638 and BRAF WT copy number ranged from 129–1391. All patients with known BRAF WT tumors had zero copies of BRAF V600E and 1380–2390 BRAF WT copies (Figure 4). Thus, there was 100% concordance between the ddPCR tumor assay and mutational status as determined by SNapShot or IHC and 100% sensitivity and specificity for the diagnosis of BRAF V600E mutation in tumor tissue.

**Figure 5:**
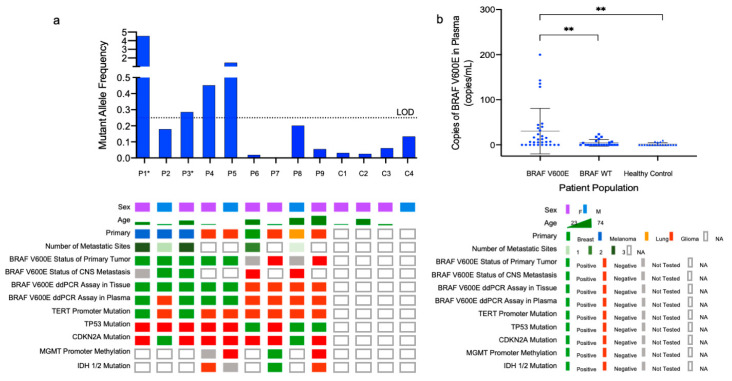
BRAF V600E and BRAF Wild Type ddPCR assay in patient plasma. (**a**) Mutant allele frequency (MAF, %), which is the ratio of BRAF V600E positive events to BRAF WT positive events, as determined by the ddPCR Plasma Assay is shown with respect to patient study IDs. The threshold for positivity, defined as the LOD, 0.285%, is shown as a dotted line. MAF ranged from 0.2–4.5% in patients with known BRAF V600E mutations (P1–5). MAF ranged from 0.0–0.2% and 0.0–0.1% in plasma from patients with known BRAF WT status (P6–9) and healthy controls (C1–4), respectively, thus all 8 known WT samples were defined as negative by the plasma assay. An OncoPrint depicting patient demographics and tumor specific information, including histopathologic testing and mutational statuses, is shown. Patients with available longitudinal samples are denoted by an (*); (**b**) BRAF V600E mutant copies per mL of grouped patient plasma is plotted against known BRAF status, as determined by the ddPCR Tumor Assay. Then, 4 µL of exNA isolated from 2 mL of plasma was used as input for the ddPCR assay with the universal primer set (ddPCR Plasma Assay). *p*-value ≤0.01 indicated by (**).

Next, we interrogated plasma samples from patients and healthy controls for BRAF V600E and BRAF WT (*n* = 5 BRAF V600E, *n* = 4 BRAF WT, and *n* = 4 healthy controls). Then, 4 µL of exNA template was used for each reaction; samples were run in replicates of 4–8 with the BRAF V600E and BRAF WT ddPCR assay with the universal primer set (i.e., ddPCR plasma assay). Patients harboring known BRAF V600E mutations had an average of 30 BRAF V600E copies/mL (4–152 BRAF V600E copies/mL), with an average of 2884 BRAF WT copies/mL (2079–3745 BRAF WT copies/mL). Patients with known BRAF WT tumors had an average of four BRAF V600E copies/mL (0–14 BRAF V600E copies/mL) and 4757 BRAF WT copies/mL (4139–6896 BRAF WT copies/mL). In healthy control plasma, an average of two BRAF V600E copies/mL (1–3 BRAF V600E copies/mL) and 3065 BRAF WT copies/mL (1868–4545 BRAF WT copies/mL). Thus, patients with known BRAF V600E mutated tumors had statistically higher copies of BRAF V600E per mL of plasma than either their BRAF WT (*p* = 0.008) or healthy control (*p* = 0.003) counterparts (Figure 5a). We called BRAF status of plasma samples based on MAF above 0.285%, the LOQ of the ddPCR plasma assay. Of patients with known BRAF V600E based on the ddPCR tumor assay, 4/5 had MAF above the LOQ, ranging from 0.286–4.544%. One patient plasma sample, P2, had a MAF of 0.179%, and thus did not meet the threshold of positivity. Among patients with known BRAF WT tumors, MAF in plasma ranged from 0.000–0.201% and in healthy controls, MAF ranged from 0.026–0.134% (Figure 5b).

### 2.4. Longitudinal Monitoring of Patients with Known BRAF V600E Mutation Status

**Figure 6:**
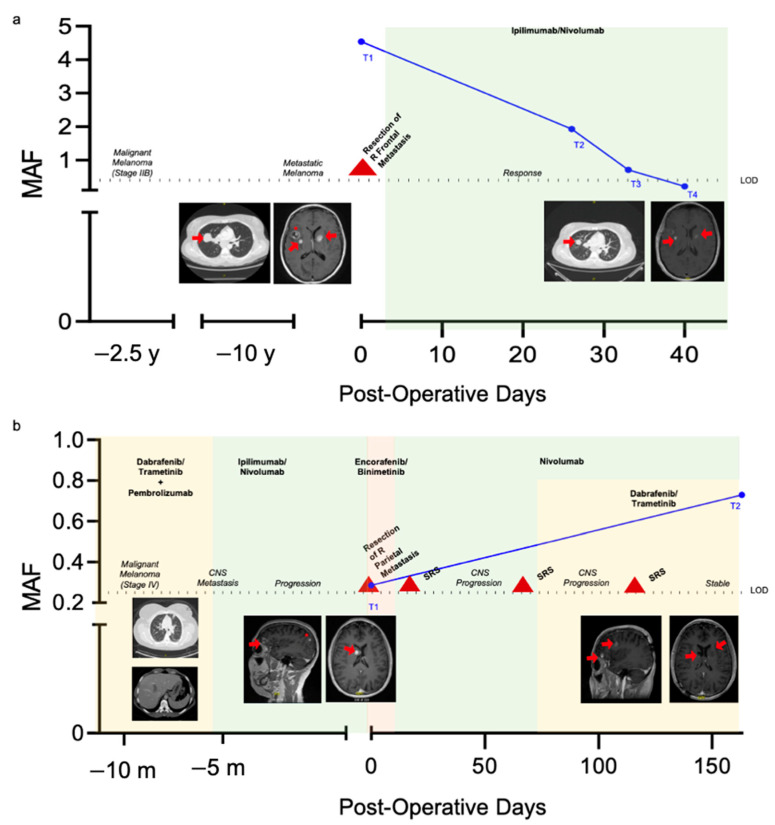
Longitudinal monitoring of BRAF V600E mutant allele frequency in patients with known BRAF V600E mutant metastatic melanoma. MAF (blue, %) as determined by the ddPCR Plasma Assay is plotted relative to post-operative days. Timepoints for the plasma assay are shown. The threshold for assay positivity, the LOD, is denoted by the dotted line. Chemotherapy regimens are signified by colored background shading and surgical and stereotactic radiosurgery (SRS) interventions are denoted by a red triangle. Intra- and extracranial metastatic foci that can be appreciated in either axial or sagittal T1 post-contrast images are highlighted with a red arrow. Longitudinal imaging of select metastasis with significant change are also shown. The CNS metastasis that was biopsied/resected is highlighted with a red star. Data for P1 is shown in (**a**) and P3 in (**b**).

Next, the ddPCR Plasma Assay was used to analyze longitudinally plasma of two patients, P1 and P3. Both patients had malignant melanoma with known BRAF V600E mutation and metastasis to the brain as well as additional extracranial metastasis, including to the abdomen and lung. BRAF V600E MAF frequency is plotted for each timepoint (T) and representative images of intra- and extracranial metastasis are shown for each patient in Figure 6. The same criteria for BRAF V600E positivity, a MAF of 0.285%, was used for analysis of longitudinal samples.

Plasma from P1 at the time of neurosurgery (T1) as well as 26 (T2), 33 (T3), and 40 (T4) days postoperatively was tested with the ddPCR plasma assay (Figure 6a). At T1, plasma was positive for the BRAF V600E mutation with a MAF of 4.544%. Clinically, the patient had been diagnosed with treatment naive, diffusely metastatic melanoma with known metastasis to the lungs and liver. The patient had seven distinct intracranial foci concerning for metastasis, three of them are seen in the first brain MRI in Figure 6a. Thus, the patient underwent neurosurgical resection of the right frontal lesion to confirm metastatic melanoma. Postoperatively, ipilimumab/nivolumab therapy was initiated. T2, which had a BRAF V600E MAF of 1.932%, was taken after one cycle of chemotherapy. One week later, another plasma sample, T3, was tested and had a MAF of 0.709%. The final timepoint, T4, was taken after the second cycle of treatment, and had a MAF of 0.215%, below the threshold of positivity. At this time, restaging scans demonstrated marked decrease in the size of the lung and abdominal metastasis as well reduction in non-resected CNS metastasis.

Plasma samples from P3 at the time of neurosurgery (T1) and 163 days postoperatively (T2) were tested with the ddPCR plasma assay (Figure 6b). Prior to the first timepoint, the patient had been diagnosed with malignant melanoma with metastasis to the lungs and abdominal cavity. They had received prior chemotherapy with multiple agents. The patient then developed intracranial lesions suspicious for metastasis, 3/6 of which can be seen in the first brain MRI in Figure 6b. This prompted surgical resection of the right parietal lesion, at which timepoint T1 was drawn. MAF at T1 was positive for the BRAF V600E mutation with a MAF of 0.286%. In the interim, the patient was started on BRAF inhibitor therapy as well as nivolumab for extracranial disease control and underwent several stereotactic radiosurgery (SRS) treatments. Despite treatment, the patient had continued progression of intracranial disease, and MAF at T2 increased to 0.729%.

## 3. Discussion

In this study, we demonstrate technical development a droplet digital PCR assay for the detection of BRAF V600E in plasma of patients with primary and metastatic brain tumors. We show the ability to detect circulating BRAF V600E in 4/5 patients with known BRAF mutant tumors in our small cohort of patients with metastatic disease, including brain metastasis (*n* = 3) and primary gliomas (*n* = 2). Furthermore, we explore the ability to detect the circulating BRAF V600E hotspot mutation for longitudinal global disease monitoring in a subset of our cohort with multifocal metastatic disease.

BRAF mutations have been identified as driver mutations in glioma subsets and extracranial cancers, including melanoma which has a high propensity to metastasize to the brain [29,30]. The advent of BRAF inhibitor therapy [24,26,31], later in combination with MEK inhibition [26,27,32], revolutionized the treatment of metastatic melanoma. Assays for the detection of BRAF mutations have thus far been developed with sensitivities between 65–89% [33,34,35]. These assays, some of which apply whole genome amplification before analysis, have limits of detection between 0.001–0.005% [34,36,37,38,39]. In comparison to our assay, those previously reported in the literature do have lower limits of detection, but when applied to clinical samples, the sensitivity of our assay is similar to those previously described. We are also able to directly analyze samples without prior whole genome amplification. Although this amplification may contribute to the lower limit of detection, in our small cohort this did not impede sensitivity. Further, our assay reduces opportunity for user variability and presents a more streamlined workflow. We also specifically compared the primer set developed by Grey et al. which detects the BRAF V600E mutation in DNA to our own DNA primer set, yielding similar BRAF copy number detection (data not shown). We additionally demonstrate that co-isolation of cfDNA and exosomal RNA with our universal primer set as compared to cfDNA alone as detected by our DNA primer set increases MAF detection as seen in Figure 2c–e [33]. These BRAF liquid biopsy assays have shown promise for prognostication and monitoring treatment response and disease progression, but they have been relatively limited in their application to CNS metastasis and primary brain tumors [40,41]. Our study adds to this existing body of literature on the application of liquid biopsy to the field on malignant melanoma with a focus on intracranial melanoma metastasis and primary brain tumors.

In metastatic melanoma, we detected BRAF V600E in P1 and P3. It is plausible P2 did not have a detectable signal because they underwent chemotherapy with BRAF and checkpoint inhibitor agents prior to neurosurgical resection of metastasis, and SRS. Additionally, the patient had stable disease at the assay timepoint. There also may have been sequestration of liquid biopsy substrates by the BBB [13,42] larger cohort of samples will be needed to determine the maximal sensitivity and specificity of this assay.

Additionally, P1 and P3 were monitored longitudinally. P1 was treatment naïve which may explain their higher MAF which decreased after initiation of checkpoint inhibitor therapy. Although previous reports by Gray et al. have suggested BRAF V600E mutation burden is less reactive to immunotherapies as compared to BRAF inhibitor therapies, our case demonstrated BRAF V600E MAF was highly reactive to checkpoint inhibition [33,43]. In contrast, P3 had worsening disease burden with an associated rise in BRAF V600E MAF. Overall, we add to an existing body of literature that demonstrates BRAF V600E levels detected in peripheral blood correlate with clinical disease status [16,33,43].

BRAF mutations have also been implicated in glioma tumorigenesis [21,44,45] prompting investigation of the use of BRAF targeted therapies in glioma [23,46,47,48,49]. In our cohort, P4 and P5 had gliomas positive for the BRAF V600E mutation with P4 having a PXA with MAF of 0.452% at the time of resection. Patients with BRAF-mutated PXA have also been shown to have improved survival as compared to their wild type counterparts, and liquid biopsy may aid in prognostication [50].

P5 was diagnosed with epithelioid GBM and was subsequently started on a clinical trial protocol with BRAF/MEK inhibitor dabrafenib/trametinib, terminated early due to compliance issues. After multiple rounds of treatments T1 was tested with our assay, revealing a MAF of 1.450%. This high MAF may be due to the progressive disease, as confirmed by their rapid clinical deterioration within 48 h of their blood draw. Notably, the MAF was higher in P5, who had a GBM, than in P4, who had a lower grade glioma, likely suggesting higher grade gliomas release a more circulating nucleic acid detectable in plasma.

Overall, our proof of principle study demonstrates the ability to detect circulating BRAF V600E hotspot mutations in a small cohort of patients with primary and metastatic brain tumors. Although this work is reflective of a small cohort of patients with primary and secondary brain tumors, future work will aim to validate the assay in larger and more homogenous cohorts. Thus, this work lays the groundwork for the ability to detect this mutation in the plasma of patients with brain tumors is important for the overall application of liquid biopsy in plasma to the CNS field, and may be useful as a diagnostic adjunct, tool for prognostication, and method for monitoring of disease and response to treatment.

## 4. Materials and Methods

### 4.1. Study Population

The study population (*n* = 13, *n* = 5 BRAF V600E, *n* = 4 BRAF wild type, *n* = 4 matched healthy controls) included 9 patients aged 18 years or older who underwent surgery at the Massachusetts General Hospital (MGH) for biopsy or resection of a primary or metastatic brain lesion and 4 matched healthy controls. Clinical BRAF status was established with either immunohistochemistry (IHC) or by the MGH SNaPShot Panel. Additional inclusion criteria for the study population included histopathological confirmation of disease and, if applicable, metastatic disease with known primary tumor. Healthy control patients with a history of oncologic, neurologic, or ongoing infectious conditions were excluded from the study. All samples were collected under IRB approved protocol no. 2017P001581 with informed patient consent. Patient demographics are depicted in oncoprint format in Figure 5a and in table format in Table 1.

### 4.2. Tumor Tissue Processing

When available, fresh tumor tissue was microdissected, suspended in RNAlater (Ambion) or flash-frozen and stored at −80 °C. In cases where fresh frozen tumor tissue was not available, FFPE slides, obtained from MGH Pathology, were used.

### 4.3. Plasma Processing

Whole blood was collected using K2 EDTA tubes with an inert gel barrier (BD Vacutainer Blood Collection Tubes). Within two hours of collection, plasma was isolated via centrifugation at 1100× *g* for 10 min at 20 °C and filtered with 0.8 µm filters. Subsequently, 1 mL aliquots of plasma were stored at −80 °C for downstream extraction and ddPCR analysis.

### 4.4. Cell Lines

The melanoma cell line, Yumel 0106 (homozygous for the BRAF V600E mutation), kindly provided by Xandra O. Breakefield, was cultured in OptiMem (Invitrogen) containing 10% fetal bovine serum (FBS) and 5% penicillin/streptomycin. The GBM cell line DBTRG-05MG (heterozygous for the BRAF V600E mutation, ATCC CRL-2020) was cultured in RPMI-1640 (ThermoFisher) containing 10% FBS and 5% penicillin/streptomycin. Human brain microvascular endothelial cells (HBMVEC, BRAF WT) were kindly provided by Xandra O. Breakefield and cultured in Microvascular Endothelial Cell Growth Medium-2 (BulletKit, Lonza) containing 10% FBS and 5% penicillin/streptomycin. gDNA and mRNA was isolated from cultures at 50–70% confluency and all cell lines are negative for mycoplasma contamination (Mycoplasma PCR Detection Kit; Applied Biological Materials).

### 4.5. DNA Isolation

gDNA was isolated from cell lines and fresh frozen tumor tissue using the DNeasy Blood and Tissue Kit (QIAgen, Germantown, MD, USA) per manufacturer’s recommendations and eluted in 200 µL of Buffer AE (QIAgen). gDNA was isolated from FFPE tissue sections using the QIAmp DNA FFPE Tissue Kit (QIAgen) per manufacturer’s recommendations and eluted in 65 µL of Buffer ATE (QIAgen). All DNA samples were assessed for concentration and purity with the NanoDrop One (ThermoFisher, Waltham, MA, USA,) and stored at −20 °C prior to downstream analysis.

### 4.6. mRNA Isolation

mRNA was isolated from cell lines using the RNeasy Mini Kit (QIAgen) per manufacturer’s recommendations and eluted in 50 µL of nuclease free water. mRNA was assessed for concentration and purity with the NanoDrop One (ThermoFisher). mRNA was then subject to reverse transcription (RT) using the SuperScript Vilo cDNA Synthesis to generate cDNA. The RT product was subsequently stored at −20 °C until ddPCR testing.

### 4.7. Cell Free DNA and Exosomal RNA Isolation

Cell free DNA (cfDNA) and exosomal RNA (exRNA) were isolated from plasma samples using the ExoLution Plus Kit (Exosome Diagnostics, a Bio-Techne brand) per manufacturer’s recommendations. cfDNA and exRNA were eluted in 20 µL of nuclease free water and assessed for concentration and purity with the NanoDrop One (ThermoFisher). Then, 14 µL of the eluate was subsequently reverse transcribed to convert exRNA to cDNA using the SuperScript Vilo cDNA Synthesis Kit (ThermoFisher). The RT product, composed of cfDNA and cDNA from the exRNA (i.e., exosomal nucleic acids, or exNA), was subsequently stored at −20 °C until ddPCR testing.

### 4.8. BRAF ddPCR Assay

Probes specific to the BRAF V600E and wild type (WT) sequence were employed, as described by Gray et al. [33]. The BRAF V600E mutation lies within exon 15 and is thus detectable on both the DNA and mRNA level. Primers were designed to test for the presence of the BRAF on the DNA level (i.e., “DNA Primer”), mRNA level (i.e., “mRNA Primer), and both the DNA and mRNA level (i.e., “Universal Primer”). Sequences for probes and primers are as follows: BRAF V600E Probe (5′-FAM-TAGCTACAGAGAAATC-MGBNFQ-3′), BRAF WT Probe (5′-VIC-CTAGCTACAGTGAAATC-MGBNFQ-3′), DNA primer forward (5′-CTACTGTTTTCCTTTACTTACTACACCTCAGA-3′) and reverse (5′-ATCCAGACAACTGTTCAAACTGATG-3′), mRNA primer forward (5′-GATATTGCACGACAGACTGCAC-3′) and reverse (5′-TCCAGACAACTGTTCAAACTGATG-3′), and Universal primer forward (5′-GACCTCACAGTAAAAATAGGTGATT-3′) and reverse (5′-AACTGTTCAAACTGATGGGACC-3′). ddPCR was performed using 10 ng of gDNA or cDNA isolated from cell lines, tumor tissue, or FFPE slides or 4 µL of exNA isolated from plasma as template, 1x ddPCR supermix (no dUTP, BioRad), 250 nM probe, and 900 nM primer. A temperature gradient was performed to determine optimal annealing temperature, and the final thermocycler conditions were as follows: 95 °C (51% ramp) for 10 min, 40 cycles of 94 °C (51% ramp) for 30 s and 62 °C for 1 min, followed by 98 °C for 10 min and held at 4 °C until analysis. Droplets were generated using the QX200 droplet generator (Bio-Rad), analyzed using the QX200 droplet reader (Bio-Rad) and data were acquired and analyzed with the QuantaSoft analysis (Bio-Rad) software.

### 4.9. Quantification of BRAF V600E Mutation in Plasma

The number of BRAF V600E and WT copies per mL of plasma was calculated from QuantaSoft data as follows: Copies/mL plasma = C EV/TV/P where C = copies per 20 µL, EV = exNA elution volume (µL), TV = exNA input into ddPCR reaction (µL) and *p* = plasma volume (mL). The BRAF V600E mutant allele frequency in plasma samples was determined by merging replicates and taking the ratio of positive BRAF V600E and BRAF WT droplets, expressed as a percent. Only samples with >10,000 droplets/well were included in the analysis.

### 4.10. dMIQE 2020 Guideline Compliance

This section of the methods describes our compliance with the updated 2020 dMIQE Guidelines for the technical development of a plasma-based ddPCR assay for the BRAF V600E mutation [51]. Specimen type numbers, sampling procedure, aliquotation, conditions and duration are provided in the following sections of the Methods: Study Population, Tumor Tissue Processing, Cell Lines, and Plasma Processing. Details about the specific Extraction techniques, Nucleic Acid Assessment/Storage, and Reverse Transcription are provided in sections: DNA Isolation, RNA Isolation, Cell Free DNA and Exosomal RNA Isolation. Information about the ddPCR Oligonucleotides and its target sequences are provided along with the protocol both in Figure 1a,b and in the section entitled BRAF ddPCR assay. Details about the assay analytical validation are provided in Figure 2 and Figure 3. Finally, information regarding data analysis is provided both in the Results, Figure 4 and Figure 5, and in the Methods section entitled Quantification of BRAF V600E Mutation in Plasma.

### 4.11. Statistical Analysis

Statistical analysis was performed using the unpaired two-tailed Student’s *t*-test in GraphPad Prism 8 software and *p* < 0.05 was considered to be statistically significant. The results are presented as mean ± SD.

## 5. Conclusions

In this study, we developed a novel ddPCR based assay for the detection of BRAF V600E in the plasma of patients with primary and metastatic brain tumors. We successfully determined BRAF mutational status in all primary tumor samples as compared to gold standard pathology. We determine BRAF mutational status with a sensitivity of 80% and specificity of 100% in the plasma of patients with brain tumors. Furthermore, we demonstrate correlation between BRAF V600E mutant allele frequency and clinical disease status in two patients with known BRAF V600E mutant melanoma. Our study demonstrates the feasibility of plasma-based brain tumor biomarker detection and paves the path for liquid biopsy based molecular profiling for diagnosis and longitudinal monitoring in patients with primary and metastatic BRAF V600E mutant brain tumors.

## Figures and Tables

**Table 1:** Baseline characteristics of individual patients.

Study ID		Age	Sex	Diagnosis	Metastatic Disease		Prior Treatment		
					Extracranial	Intracranial	NSGY	Chemotherapy	SRS
BRAF V600E	P1	43	F	Malignant Melanoma	Lung, Abd	7	-	-	-
	P2	30	M	Malignant Melanoma	NA	8	x2	Yes *	Yes
	P3	49	F	Malignant Melanoma	Lung, Abd	6	-	Yes *	-
	P4	30	F	Pleomorphic Xanthoastrocytoma	NA	NA	-	-	-
	P5	23	M	Epithelioid GBM	NA	NA	GTR	Yes *	Yes
BRAF WT	P6	52	F	Invasive Ductal Carcinoma, Breast	Lung, Liver	9	None	Yes	Yes
	P7	31	F	GBM	NA	NA	GTR	Yes	Yes
	P8	62	M	Lung Adenocarcinoma	Abd	1	None	-	-
	P9	74	F	GBM	NA	NA	Biopsy	-	-

Prior chemotherapy treatments with BRAF/MEK inhibitors are denoted by an (*). Abd = abdomen, LN = lymph node, GBM = glioblastoma, NSGY = neurosurgery, GTR = gross total resection, SRS = stereotactic radiosurgery, “-” = none, NA = not applicable.

## Data Availability

The data presented in this study are available on request from the corresponding author. The raw ddPCR runs are not publicly available because raw PCR runs are not commonly shared but they are available upon request.
